# Node centrality measures are a poor substitute for causal inference

**DOI:** 10.1038/s41598-019-43033-9

**Published:** 2019-05-02

**Authors:** Fabian Dablander, Max Hinne

**Affiliations:** Department of Psychological Methods, University of Amsterdam, Amsterdam, Netherlands

**Keywords:** Network models, Human behaviour

## Abstract

Network models have become a valuable tool in making sense of a diverse range of social, biological, and information systems. These models marry graph and probability theory to visualize, understand, and interpret variables and their relations as nodes and edges in a graph. Many applications of network models rely on undirected graphs in which the absence of an edge between two nodes encodes conditional independence between the corresponding variables. To gauge the importance of nodes in such a network, various node centrality measures have become widely used, especially in psychology and neuroscience. It is intuitive to interpret nodes with high centrality measures as being important in a causal sense. Using the causal framework based on directed acyclic graphs (DAGs), we show that the relation between causal influence and node centrality measures is not straightforward. In particular, the correlation between causal influence and several node centrality measures is weak, except for eigenvector centrality. Our results provide a cautionary tale: if the underlying real-world system can be modeled as a DAG, but researchers interpret nodes with high centrality as causally important, then this may result in sub-optimal interventions.

## Introduction

In the last two decades, network analysis has become increasingly popular across many disciplines dealing with a diverse range of social, biological, and information systems. From a network perspective, variables and their interactions are considered nodes and edges in a graph. Throughout this paper, we illustrate the network paradigm with two areas where it has been applied extensively: psychology and neuroscience. In psychology, particularly in psychometrics^1^ and psychopathology^2,3^, networks constitute a shift in perspective in how we view psychological constructs. Taking depression as an example, the dominant latent variable perspective assumes a latent variable ‘depression’ causing symptoms such as fatigue and sleep problems^4^. Within the network perspective, there is no such latent variable ‘depression’; instead, what we call depression arises out of the causal interactions between its symptoms^5^. Similarly, the network paradigm is widely used in neuroscience. Here, networks describe the interactions between spatially segregated regions of the brain^6,7^. It has been shown that alterations in brain network structure can be predictive of disorders and cognitive ability^8–10^ which has prompted researchers to identify which aspects of network structure can explain these behavioural variables^11–16^.

A principal application of network theory is to gauge the importance of individual nodes in a network. Here, we define the importance of a node as the extent to which manipulating it alters the functioning of the network. The goal in practical applications is to change the behaviour associated with the network in some desired way^13–21^. A number of measures, commonly referred to as centrality measures, have been proposed to quantify this relative importance of nodes in a network^22–24^. Colloquially, importance is easily confused with *having causal influence*. However, this does not follow from the network paradigm (that is, not without additional effort through e.g. randomized trials), as it provides only a statistical representation of the underlying system, not a causal one; although one might study how the system evolves over time and take the predictive performance of a node as its Granger-causal effect^25,26^. If we want to move beyond this purely statistical description towards a causal, and eventually mechanistic one (which we need to fully understand these systems), we require additional assumptions. With these, it is possible to define a calculus for *causal inference*^27,28^. Although it is not always clear whether the additional assumptions of this framework are warranted in actual systems^29,30^, without it we lack a precise way to reason about causal influence. In this paper, we explore to what extent, if at all, node centrality measures can serve as a proxy for causal inference.

The paper is organized as follows. In Section 2, we discuss the necessary concepts of network theory and causal inference. Next, in Section 2.5, we discuss how these two different perspectives are related. In Section 3 we describe our simulations, and discuss the results in Section 4. We end in Section 5 with a short summary and a note on the generalizability of our findings.

## Preliminaries

### Undirected networks

The central objects in network theory are graphs. A graph ${\mathscr{G}}=(V,E)$ consists of nodes *V* and edges *E* connecting those nodes; nodes may be for instance neuronal populations or symptoms, while edges indicate whether (in unweighted networks) or how strongly (in weighted networks) those nodes are connected. There are several ways of defining the edges in a graph. A common approach is to assume that the data come from a Gaussian distribution and to compute for each pair of nodes their partial correlation, which serves as the strength of the corresponding edge. The absence of an edge between a pair of nodes (i.e., a partial correlation of zero) indicates that these nodes are *conditionally independent*, given the rest of the network. Such networks, referred to as *Markov random fields* (MRFs)^31,32^, capture only symmetric relationships.

#### Node centrality measures

Markov random fields can be studied in several ways. Originating from the analysis of social networks^22^, various centrality measures have been developed to gauge the importance of nodes in a network^33^. A popular measure is the *degree* of a node which counts the number of connections the node has^22^. A generalization of this measure takes into account the (absolute) *strength* of those connections^23^. Other measures, such as *closeness* and *betweenness* of a node, are not directly based on a single node’s connection, but on shortest paths between nodes^34^, or, such as *eigenvector centrality*, weigh the contributions from connections to nodes that are themselves central more heavily^35^ (see Appendix for mathematical details).

### Causal inference background

Causal inference is a burgeoning field that goes beyond mere statistical associations and informs building models of the world that encode important structural relations and thus generalize to new situations^36–39^. One widespread formalization of causal inference is the *graphical models framework* developed by Pearl and others^27,40^, which draws from the rich literature on structural equation modeling and path analysis^41^. In contrast to Markov random fields, the causal inference framework used here relies on *directed acyclic graphs* (DAGs), which do encode directionality. Assumptions are required to represent the independence relations in data with graphs, and further assumptions are needed to endow a DAG with causal interpretation^29,42^. We briefly discuss these assumptions below, and refer the reader for more in-depth treatment to excellent recent textbooks on this topic^27,28,43,44^.

### Causal inference with graphical models

Consider the following example. We know from biology that storks do not deliver human babies, and yet there is a strong empirical correlation between the number of storks (*X*) and the number of babies delivered (*Y*)^45^. In an attempt to resolve this, textbook authors usually introduce a third variable *Z*, say economic development, and observe that *X* is *conditionally independent* of *Y* given *Z*, written as *X* ⫫ *Y*|*Z*^46^. This means that if we fix *Z* to some value, there is no association between *X* and *Y*. Using this example, we introduce the first two rungs of the ‘causal ladder’^39,47^, corresponding metaphorically to the process of *seeing* and *doing*^42^.

#### Seeing

**Figure 1:**
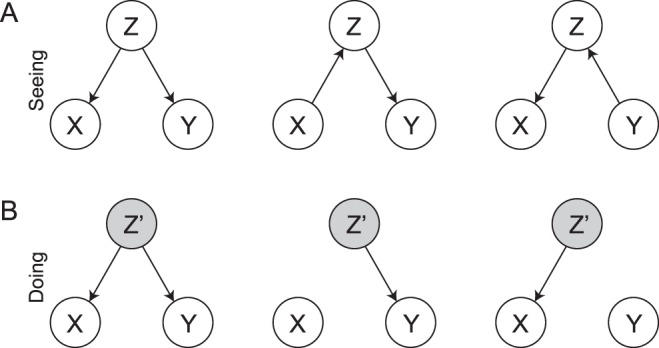
(**A**) *Seeing*: Three *Markov equivalent* DAGs encoding the conditional independence structure between *number of storks* (*X*), *number of delivered babies* (*Y*), and *economic development* (*Z*) (see main text). (**B**) *Doing*: How the DAG structure changes when intervening on *Z*, i.e., *do*(*Z* = *z*′).

DAGs provide a means to visualize conditional independencies. This can be especially helpful when analyzing many variables. Figure 1A displays the three possible DAGs corresponding to the stork example–all three encode the fact that *X* ⫫ *Y*|*Z*. To see this, we require a graphical independence model known as ‘*d*-separation’^48–50^, which helps us read off conditional independencies from a DAG. To understand *d*-separation, we need the concepts of a *walk*, a *conditioning set*, and a *collider*.

- A walk *ω* from *X* to *Y* is a sequence of nodes and edges such that the start and end nodes are given by *X* and *Y*, respectively. In the middle DAG in Fig. 1A, the only possible walk from *X* to *Y* is (*X* → *Z* → *Y*).
- A conditioning set ${\mathcal L}$ is a set of nodes corresponding to variables on which we condition; note that it can be empty.
- A node *W* is a collider on a walk *ω* in ${\mathscr{G}}$ if two arrowheads on the walk meet in *W*; for example, *W* is a collider on the walk (*X* → *W* ← *Y*). Conditioning on a collider means to *unblock* a path from *X* to *Y* which would have been blocked without conditioning on the collider.

Using these definitions, two nodes *X* and *Y* in a DAG ${\mathscr{G}}$ are *d*-separated by nodes in the conditioning set ${\mathcal L}$ if and only if members of ${\mathcal L}$ block all walks between *X* and *Y*. Because *Z* is not a collider in any of the DAGs displayed in Fig. 1A, *Z d*-separates *X* and *Y* in all three graphs. Graphs that encode exactly the same conditional independencies are called *Markov equivalent*^31^. We now have a graphical independence model, given by *d*-separation, and a probabilistic independence model, given by probability theory. In order to connect the two, we require the *causal Markov condition* and *faithfulness*, which we will discuss in the next section. If one is merely concerned with association–or ‘seeing’–then the three DAGs in Fig. 1 are equivalent; the arrows of the edges do not have a substantive interpretation or, as Dawid (2010a, p. 66)^29^ puts it, ‘are incidental construction features supporting the *d*-separation semantics.’ Below, we go beyond ‘seeing’.

#### Doing

It is perfectly reasonable to view DAGs as only encoding conditional independence. However, merely seeing the conditional independence structure in our stork example does not resolve the puzzle of what causes what. In an alternative interpretation of DAGs, the arrows encode the flow of information and causal influence–Hernán & Robins (2006) call such DAGs *causal*^51^. For example, the rightmost DAG in Fig. 1 models a world in which the number of delivered babies influences economic development which in turn influences the number of storks. In contrast, and possibly more reasonable, the DAG on the left describes a *common cause* scenario in which the number of storks and the number of babies delivered do not influence each other, but are related by economic development; e.g., under a flourishing economy both numbers go up, inducing a spurious correlation between them. In order to connect this notion of causality with conditional independence, we need the following two assumptions.

*Assumption I: Causal Markov Condition*. We say that a graph ${\mathscr{G}}$ is causally Markov to a distribution ${\mathscr{P}}$ over *n* random variables (*X*_1_, *X*_2_, …, *X*_*n*_) if we have the following factorization:

1 $$P({X}_{1},\ldots ,{X}_{n})=\prod _{i=1}^{n}P({X}_{i}| {\bf{p}}{{\bf{a}}}_{{\bf{i}}}^{{\mathscr{G}}})\,,$$

where ${\bf{p}}{{\bf{a}}}_{{\rm{i}}}^{{\mathscr{G}}}$ are the *parents* of node *X*_*i*_ in ${\mathscr{G}}$^27,28,52^. This states that a variable *X*_*i*_ is conditionally independent of its non-effects (*descendants*), given its direct causes (parents). When (2) holds we can derive conditional independence statements from causal assumptions–a key step in testing models.

*Assumption II: Faithfulness*. We say that a distribution ${\mathscr{P}}$ is *faithful* to a graph ${\mathscr{G}}$ if for all disjoint subsets *X*, *Y*, *Z* of all nodes *V* it holds that:2where  is the independence model implied by the distribution, and  is the independence model implied by the graph^28,53^. In other words, while the causal Markov condition allows us to derive probabilistic conditional independence statements from the graph, faithfulness implies that there are no further such conditional independence statements beyond what the graph implies.

This causal DAG interpretation gives causal inference an *interventionist* flavour. Specifically, in the leftmost DAG, if we were to change the number of babies delivered, then this would influence the number of storks. In contrast, when doing the same intervention on the right DAG, the number of storks would remain unaffected. Pearl developed an algebraic framework called *do*-calculus to formalize this idea of an intervention^43^. In addition to conditional probability statements such as *P*(*Y*|*X* = *x*) which describes how the distribution of *Y* changes when *X* is observed to equal *x*, the *do*-calculus admits statements such as *P*(*Y*|*do*(*X* = *x*)) which describes how the distribution of *Y* changes under the hypothetical of setting *X* to *x*. The latter semantics are usually associated with experiments in which we assign participants randomly to *X* = *x*. Pearl showed that, given the assumptions of causal DAGs, such statements can also be valid for observational data. If possible, such causal claims can further be tested using experiments.

Intervening on a variable changes the observed distribution into an *intervention distribution*^54^. As an example, setting *Z* to *z*′, i.e., *do*(*Z* = *z*′), cuts the dependency of *Z* on its parents in the causal DAG (since it is intervened on and therefore by definition cannot be caused by anything else), and allows us to see how other variables change as a function of *Z*; see Fig. 1B.

### Measuring causal influence

The *do*-calculus and the intervention distribution allow us to define a measure of causal influence called the *average causal effect* (ACE)^27^ as

3 $$ACE(Z\to Y)={\mathbb{E}}[Y| do(Z=z+1))]-{\mathbb{E}}[Y| do(Z=z)]\,,$$

where the expectation is with respect to the intervention distribution. The ACE has a straightforward interpretation which follows directly from its definition, namely that it captures the differences in the expectation of *Y* given *Z* when *Z* is forced to a particular value. For each node in the DAG, we compute the *total ACE*, that is, the sum of its absolute average causal effect on its children, i.e.,

4 $$AC{E}_{{\rm{t}}{\rm{o}}{\rm{t}}{\rm{a}}{\rm{l}}}({X}_{j})=\sum _{i\in Ch({X}_{j})}|ACE({X}_{j}\to {X}_{i})|\,,$$

where *Ch*(*X*)_*j*_ denotes the direct children of *X*_*j*_.

Janzing *et al*.^54^ pointed out that the ACE measure has a number of deficits; for example, it only accounts for linear dependencies between cause and effect. To address these and others, they propose an alternative measure which defines the causal strength of one arrow connecting two nodes as the Kullback-Leibler (KL) divergence between the observational distribution *P*–which corresponds to the DAG before the intervention–and the intervention distribution which results when ‘destroying’ that particular arrow, *P*_*S*_^54^. In the case of multivariate Gaussian distributions, this becomes

5 $$C{E}_{KL}(Z\to Y)=KL(P\parallel {P}_{S})=\frac{1}{2}({\rm{t}}{\rm{r}}[{{\rm{\Sigma }}}_{S}^{-1}{\rm{\Sigma }}]-\,{\rm{l}}{\rm{o}}{\rm{g}}\,\frac{|{\rm{\Sigma }}|}{|{{\rm{\Sigma }}}_{S}|}-n)\,,$$

where Σ and Σ_*S*_ are the covariance matrices of the respective multivariate Gaussian distributions, and *n* is the number of nodes. By ‘destroying’ an arrow *Z* → *Y*, we mean to remove the causal dependency of *Y* on *Z*, and instead feeding *Y* with the marginal distribution of *Z*^54^. For linear Gaussian systems and nodes with independent parents, ACE and *CE*_*KL*_ are mathematically very similar^54^. To calculate the *total* KL-based causal effect of a node, we remove all its outgoing edges. In the remainder, we let *CE*_*KL*_ refer to this total effect. Note that *CE*_*KL*_ is measured in *bits* which is less intuitive than *ACE* which resembles linear regression.

### Relating directed to undirected networks

**Figure 2:**
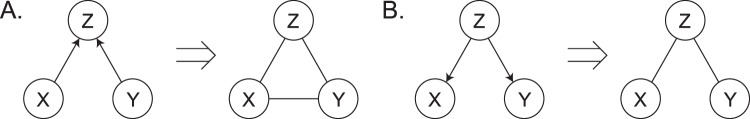
(**A**) Shows how an underlying directed acyclic graph (left) relates to an undirected graph (right) by dropping the arrows and inducing dependency between parents. While the mapping between a DAG and its moral graph is one-to-one, the resulting undirected network has six Markov equivalent DAGs. (**B**) The stork example. Conditioned on economic development (*Z*), the number of storks (*X*) and the number of babies born (*Y*) are independent.

As we saw earlier, both the Markov random field and the directed acyclic graph represent conditional independencies. In fact, it is straightforward to first look for collider structures–also called *v*-structures–in the DAG, where pairs of nodes both points toward the same child node. In this case, we construct an MRF from a DAG by connecting these for every pair of parents of each child, and then dropping the arrowheads. This procedure follows from *d*-separation (see Section 2): recall that in the MRF, an edge represents conditional dependence, given all the other nodes. So if two parent nodes are marginally independent in the DAG, they become dependent once we condition on their child node. An example of this process is shown in Fig. 2A. The resulting undirected graph is also called the *moral graph* of the particular DAG^31^. If in our stork example we assume that indeed economic development drives both the number of babies as well as the number of storks, we do not have a *v*-structure. In this case, transforming the DAG into an MRF does not involve any marrying of parent nodes, and the DAG is obtained by simply dropping the arrowheads (see Fig. 2B).

Information is lost when transforming the conditional independencies captured in a DAG to a MRF–without contextual constraints we cannot uniquely determine the true DAG from a MRF. This is due to three reasons. First and most obviously, by discarding the arrowheads we do not know whether, for example, *X*  → *Z* or *Z* →*X*, but only that *X* − *Z* which creates a symmetry where none was before. Consider again Fig. 2A. Here, node *Z* does not have any causal effect, but in the moral graph it is indistinguishable from the other nodes. Consequently, where there was a difference between these variables in the DAG, this difference is now lost. Second, additional edges are introduced when there are collider nodes (i.e., the child nodes in *v*-structures). In our example, this implies the edge *X* −    *Y*, which was not present in the DAG. Third, an undirected network may map to several Markov equivalent DAGs. That is, there may be multiple DAGs (each with their own specific distributions of causal effects) that, through the process described above, result in the same MRF^55^. Despite these fundamental differences between DAGs and MRFs, some information regarding the causal structure of the DAG might remain after the transformation into a MRF. For example, a node with many effects in the DAG may still be a highly connected node in the resulting MRF, which could be identified with a centrality measure. In the following simulation, we investigate to which extent the ranking of causal influence can be recovered from the Markov random field.

## Simulation Study

### Generating data from a directed network

**Figure 3:**
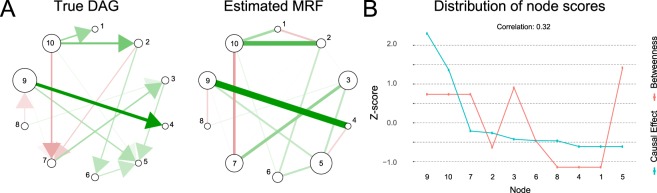
(**A**) Shows the true directed causal structure and the estimated undirected network for *v* = 10 nodes, a network density of *d* = 0.3, and *n* = 1000 observations. Node size is a function of the (z-standardized) causal effect (left) or betweenness centrality (right). (**B**) Shows the distributions of the causal effect scores *CE*_*KL*_ and betweenness centrality *C*_*B*_ for the ten nodes in the example. Nodes are ordered by causal effect. In this example, the rank-correlation between the causal effects and betweenness centrality is 0.32. As our simulations show, this is a prototypical case. An interactive version of this plot is available as a R Shiny app at https://fdabl.shinyapps.io/causality-centrality-app/.

A DAG specifies a system of equations which is called a structural equation or structural causal model^28^. We assume that the variables follow a linear structural causal model with Gaussian additive noise. In a DAG, due to the causal Markov condition, each variable *X*_*i*_’s value is a (linear) function of its parents, which themselves are (linear) functions of their parents, and so on. This regress stops at root nodes, which have no parents. The values of the children of the root nodes, and the children of their children and so on, follow from the specific functional relation to their parents–they ‘listen to’ them–and additive noise. Thus, generating data from a DAG means generating data from a system of equations given by the structural causal model. For example, assuming only one root node *X*_*R*_, we have *X*_*R*_ = *N*_*R*_ and ${X}_{i}=f({\bf{p}}{{\bf{a}}}_{{\bf{i}}}^{{\mathscr{G}}})+{N}_{i}$ for all nodes *i*, where *N*_*R*_ and *N*_*i*_ are independent noise variables following a standard normal distribution. For our simulation, we assume that *f*(⋅) is simply a linear combination of the values of the parent nodes, where the weights are drawn from a normal distribution with mean 0.3 and standard deviation 0.5; in the Appendix we illustrate how a different choice affects our results. For each DAG, we then estimate its undirected network using the graphical lasso^56,57^, and proceed to compute five different node centrality measures: degree, strength, closeness, betweenness, and eigenvector centrality (see also Section 2.1). We calculate the node-wise *CE*_*KL*_ and *ACE*_total_ scores based on the true DAG. We repeat each configuration (see below) 500 times, and compute rank-based correlations between *CE*_*KL*_ and all other measures. Note that our results do not change if we correlate the centrality measures with ACE_total_ instead of CE_KL_. Figure 3 displays what a particular simulation repetition might look like. Note how, for example, node 9 is highly influential in the causal structure, but node 5 is more central in the MRF. Readers who want to explore the relation between centrality and causal measures interactively are encouraged to visit https://fdabl.shinyapps.io/causality-centrality-app/.

### Types of networks

**Figure 4:**
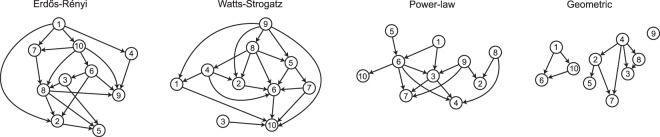
Examples of the generated directed acyclic graphs that are used as ground truth in the simulations.

The topology of a network affects its distribution of centrality scores. Because of this, we run our simulation procedure for four common network models: the Erdős-Rényi (ER) model, the Watts-Strogatz (WS) model, power-law (PL) networks, and geometric (Geo) networks. In the ER model, networks are generated by adding connections independently at random for each pair of nodes^58^. ER networks are characterized by relatively short path-lengths and low clustering. In contrast, the WS networks have the so-called ‘small-world’ property, which indicates that these networks have both short-path lengths as well as high clustering^59^. The PL networks are networks where its degree distribution follows a power-law distribution^60^. This implies that a few nodes have many connections, while most nodes are only sparsely connected to the rest of the network. These networks can be generated for instance through preferential attachment. Here, at each successive step a new node is added to the network, which is connected to the already existing nodes with a probability proportional to their degree. The final network type we consider, Geo, assumes that the nodes of the network are embedded in some metric space, and that the probability of a connection between the nodes is inversely proportional to the distance between them^61^. These networks may serve as a simple model for networks that are constrained by a physical medium, such as brain networks. They also allow isolated communities of nodes to emerge because the probability of connection between nodes far away from each other goes to zero; see Fig. 4. For each of the four network types, we randomly generate a DAG with number of nodes *v* = {10, 20, 30, 40, 50}, network density *d* = {0.1, 0.2, …, 0.9}, and then generate *n* = 5000 observations from that DAG. The density of a directed acyclic graph is given by *d* = *m*/(*v*(*v* − 1)), where *m* is the number of directed connections.

## Results

**Figure 5:**
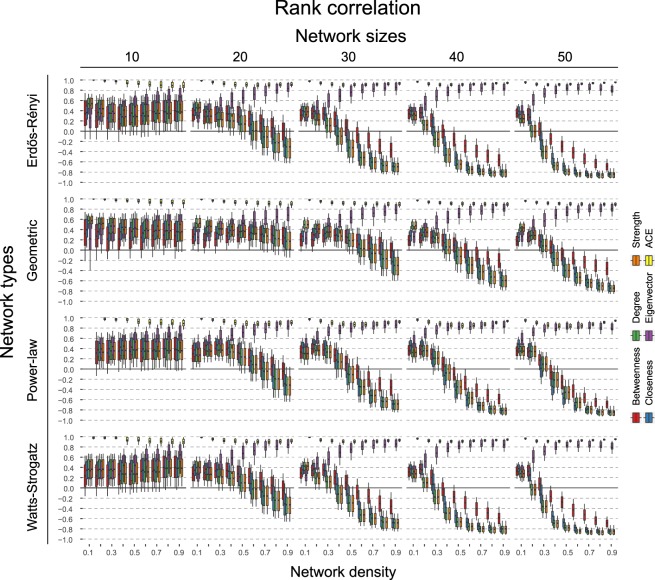
Shows the rank-based correlation *r*_*s*_ of the information theoretic causal effect measure *CE*_*KL*_ with other measures across varying types of networks, number of nodes, and connectivity levels. Error bars denote 10% and 90% quantiles across the 500 simulations. Black lines indicate the zero correlation level.

**Figure 6:**
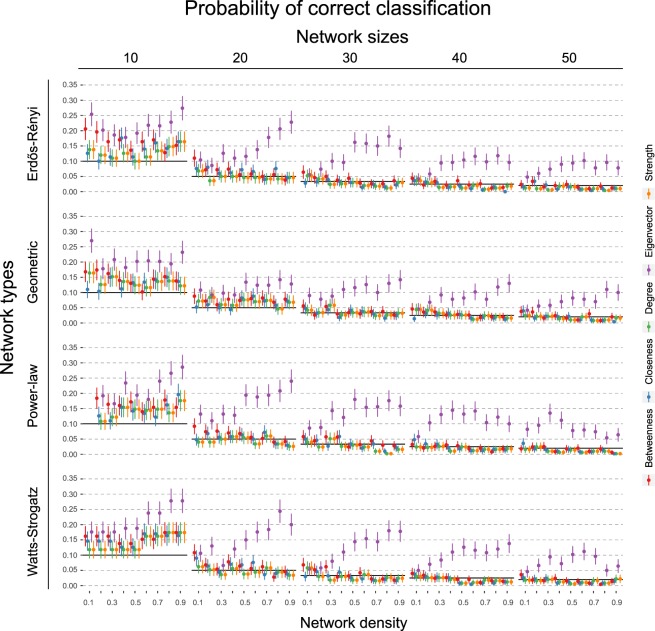
Shows the probability of choosing the node with the highest causal effect when using the node with the highest respective centrality measure. Black horizontal lines indicate chance level. Error bars denote 95% confidence intervals across the 500 simulations.

Figures 5 and 6 display the result of the simulation study. Figure 5 shows a homogeneous picture of correlation across the types of graphs: all centrality measures show a mean rank-correlation between 0.3 and 0.5, with 10–90% quantiles between −0.1 and 0.7. This correlation decreases and becomes stronger negative for graphs of increasing size (30 nodes or more), as a function of network density. It is impossible to generate a power-law distributed graph that is both very small and sparse. These cases are therefore omitted in the results in Figs 5 and 6. Note that the large variability in smaller graphs is partly because the number of data points to compute the correlation, i.e. the number of nodes, is small, and this variability decreases with the size of the graph.

If we think in terms of interventions, then we may seek to find the node which has the highest causal influence in the system. Figure 6 illustrates how often we correctly identify this node when basing our decision on centrality measures, compared to picking a node at random. The 95% confidence intervals cross the chance baseline in the majority of cases, indicating that choosing which node to intervene on based on centrality measures is not better than selecting a node at random for finding the most important node according to the DAG.

Interestingly, the exception to these results is eigenvector centrality. As graph size increases, this centrality measure shows the reverse trend compared to the other measures: as the network density increases, the correlation with the causal effect actually increases. Naturally, this also improves the classification performance when identifying the top node. Qualitatively, we observe few differences between the different graph topologies. On some level, this is not surprising, as the steps to go from DAGs to undirected networks remain the same (see Section 2.5). In general, we observe that the correlation between the measures dampens more slowly for the Geometric network as the number of nodes increase. We explain this together with the negative correlation and the unique pattern of eigenvector centrality below.

### Interpretation of key results

A possible explanation for the different trends of the centrality measures as the network density increases, is as follows. As a preliminary, note that by dropping the directionality, the undirected network cannot distinguish between root and leaf nodes. Because a DAG can be written as an ordered system of equations (this is also how one generates data from a DAG, see Section 3), the more nodes there are, the more pronounced the influence of a few top nodes as they have more children. This is the case to a smaller extent in Geometric networks, as here different hubs of nodes can emerge (see Fig. 4), which explains the slower trend for these networks. Furthermore, when ordering the nodes along causal effect, the distribution naturally becomes right-skewed (see Fig. 3). In contrast, the distribution over the centrality measures becomes left-skewed. This is because leaf nodes can have multiple parents which, when married, bump up the nodes’ centrality score; a similar thing cannot happen for root nodes, as they do not have parents. This process is a function of the network density. In sparse networks (e.g., *d* = 0.1), leaf nodes are much less likely to have many parents, and so this effect dissipates. With increased network density, leaf nodes have more parents, and marrying them increases their centrality scores more, relative to the scores of the root nodes. This left-skew is especially present for ‘local’ measures such as node degree and strength, as they only take into account the direct neighbours of a node. In contrast, the left-skew and thus negative correlation is less pronounced for closeness and especially betweenness, as they take into account ‘global’ information.

The fact that eigenvector centrality does predict the node with the highest causal influence and tracks the causal ranking reasonably well, is presumably due to the following. By transforming the DAG into a MRF, we increase the degree of those nodes that become married. But as eigenvector centrality considers the transitive importance of nodes (i.e., nodes that have many important neighbours become themselves important), this increased weight further down the original DAG structure is propagated back to those nodes higher up, which are the causally important variables. For other measures, such as betweenness or closeness, the additional paths created by transforming into a MRF can make a node more central, but this effect does not propagate back to other nodes. Implicitly, eigenvector centrality thus helps preserve the ordering of nodes from top to bottom in the DAG, which explains the positive correlation. In the next section, we summarize and discuss the generalizability of our results.

## Discussion

It is difficult to formulate a complete theory for a complex multivariate system, such as the interacting variables giving rise to a psychological disorder, or information integration between remote regions of the brain. Network models have proven a valuable tool in visualization, interpretation, and the formulation of hypotheses in systems such as these. One popular way of analyzing these networks is to compute centrality measures that quantify the relative importance of the nodes of the networks, which may in turn be used to inform interventions^62–66^. For example, in a network of interacting variables related to depression, the intuition is that intervening on highly central variables affects the disorder in a predictable way^21,67,68^. Implicitly, this suggests that these nodes have interesting *causal* effects.

In this paper, we have examined the extent to which different centrality measures (degree, strength, closeness, betweenness, and eigenvector) can actually recover the underlying causal interactions, as represented by a directed acyclic graph (DAG). (One may, of course, engage in learning the causal structure directly^69,70^). Using simulations, we found that the rank correlation between nodes ranked by centrality measure and nodes ranked by causal effect is only moderate, and becomes negative with increased network size and density. If the goal is to select the node with the highest influence, then selecting the node with the highest centrality score is not better than choosing a node at random. This holds for the majority of graphs and network densities studied here. A notable exception is eigenvector centrality, which, by considering the transitive importance of nodes, shows a strong positive correlation across all settings (see Section 4.1). Note that the results change slightly when generating data from a graph where all edges have zero mean (see the Appendix). However, this setting is unrealistic in empirical contexts.

### Limitations–is the world a DAG

The causal inference framework based on DAGs discussed here provides an elegant and powerful theory of causality^27,28^ (although it should be noted that alternative operationalizations exist^30,71^). It is closely related to the notion of *intervention* (as described in Section 2.3.2), the idea that we can alter the behaviour of a system by setting certain variables to a particular value. Intuitively, we often think about Markov random fields (MRFs), i.e. networks of (in)dependencies between variables, in the same way. However, DAGs and MRFs describe different views on a dynamical system. Here, by generating data from a known DAG and then applying network centrality analyses to MRF estimated from these data, we find that centrality measures correlate only poorly with measures of causal influence. The extent to which this finding generalizes to real world applications depends on whether the fundamental assumptions of causal inference, the *causal Markov condition* and *faithfulness*, hold in practice (see Section 2.3.2). The former encodes that, given the parents of a node, the node is independent of all its non-descendants (i.e. all nodes further up in the DAG). This idea allows for targeted interventions^28^. Faithfulness on the other hand implies that there are no additional independencies in the distribution of the data other than those encoded by the DAG. As it turns out, these assumptions may not hold in practice.

For example, in fMRI data which is used in many network neuroscience studies, the neuronal signal is actually convolved by a response function that depends on local haemodynamics^72^. This may cause the causal Markov assumption to be violated. Furthermore, these studies typically measure only regions in the cortex, so that the DAG may in fact miss common causes from other relevant structures that reside in the brain stem. In EEG/MEG, the temporal resolution is much better, but it is difficult to reconstruct where exactly the measured signal came from^73^. The fundamental question of what we take to be nodes in our graphs is therefore not trivial.

Similar problems arise in network psychometrics. For instance, when using DAGs to analyze item level data, we may be unable to distinguish between *X* causing *Y* or *X* simply having high conceptual overlap with *Y*^72^. A potential solution is to re-introduce latent variables into the network approach, and model causal effects on the latent level^74^. Furthermore, in response to Danks *et al*.^75^, Cramer *et al*.^76^ argue that causal discovery methods are inappropriate for psychological data for two reasons. First, these methods assume that individuals have the same causal structure. This is a fundamental issue (also in neuroscience)^77^, but is outside the scope of our paper. Second, DAGs do not allow cycles (i.e., they do not model feedback), which is why others propose to focus on modelling time-series data instead^78^. To pursue this further, it would be interesting to compare measures of causal influence with predictive power from time-series analysis models–essentially, Granger causality^25^. However, it is easy to construct examples detailing where predictive power does not imply causal influence^28^.

While DAGs do not allow feedback loops, one may argue that at a sufficiently high temporal-resolution no system needs to have cycles. Instead, the system is essentially described by a DAG at each discrete unit of time. Only once we aggregate multiple time instances into a coarser temporal resolution, those DAGs become superimposed, suggesting cyclic interactions. This idea may be modelled with a mixture of DAGs so that the causal structure can change over time^79^, which would be applicable to longitudinal studies. Another approach to deal with cycles is to assume that the system measured is in its equilibrium state, and use directed cyclic graphical models to model interactions between variables^80–82^. As abstractions of the underlying dynamical system, causal graphical models are therefore still useful; they provide readily interpretable ways of analyzing the effect of an intervention^83,84^.

In general, if we want to do justice to the temporal character of these data, both brain networks as well as networks of psychological disorders may best be described by (nonlinear) dynamical systems theory^85–87^. While the mapping between causal influence in a DAG and node centrality measures in a MRF may be poor, node centrality measures might predict the underlying dynamical system in a more meaningful way. We believe that this is an interesting direction that can be tackled by simulating data from a dynamical system and estimating both a DAG and a MRF from these data. However, this requires a notion of variable importance on the level of the dynamical system, which is not trivial to construct.

## Conclusion

In network models, the relative importance of variables can be estimated using centrality measures. Once important nodes are identified, it seems intuitive that if we were to manipulate these nodes, the functioning of the network would change. The consequence of this is that one easily attributes causal qualities to these variables, while in fact we still have only a statistical description.

In this paper, we analyzed to what extent the causal influence from the original causal structure can be recovered using these centrality measures, in spite of the known differences between the two approaches. We find that indeed centrality measures are a poor substitute for causal influence, although this depends on the number of nodes and edges in the network, as well as the type of centrality measure. This should serve as a note of caution when researchers interpret network models. Until a definitive theory of causation in complex systems becomes available, one must simply make a pragmatic decision of which toolset to use. However, we should be careful not to confuse concepts from one framework with concepts from the other.

## Centrality Measures

The following three centrality measures are based on^23^ who generalize the centrality measures proposed by^22^ for binary networks.

*Degree* of a node gives the number of connections the node has. Let *w*_*ij*_ be the (*i*, *j*)^th^ entry of the (weighted) adjacency matrix *W* describing the network. We define the degree of node *x*_*i*_ as

6 $${C}_{D}({x}_{i})=\sum _{j=1}^{n}{\mathbb{1}}({w}_{ij}\ne 0)\,,$$

where ${\mathbb{1}}$ is the indicator function.

*Node strength* is a generalization of *degree* for weighted networks, defined for node *x*_*i*_ as

7 $${C}_{S}({x}_{i})=\sum _{j=1}^{n}|{w}_{ij}|\,,$$

where *n* is the number of nodes and |·| the absolute value function.

The *closeness* and *betweenness* a node *x*_*i*_ are based on shortest paths. Denote the cost of travelling from node *x*_*i*_ to *x*_*j*_ be the inverse of the weight of the edge connecting the two nodes. Then, define the shortest path between two nodes *x*_*i*_ and *x*_*j*_, *d*(*i*, *j*), as the path which minimizes the cost of travelling from *x*_*i*_ to *x*_*j*_. With this, we can define the closeness of a node *x*_*i*_ as

8 $${C}_{C}({x}_{i})={[\sum _{j=1}^{n}d(i,j)]}^{-1},$$

and betweenness as

9 $${C}_{B}({x}_{i})=\sum _{j=1}^{n}\sum _{k=1}^{N}\frac{{g}_{jk}({x}_{i})}{{g}_{jk}}\,,$$

where *g*_*jk*_ is the number of shortest paths between *x*_*j*_ and *x*_*k*_, and *g*_*jk*_(*x*_*i*_) is the number of those paths that go through *x*_*i*_. The shortest paths are usually found using Dijkstra’s algorithm^88^.

*Eigenvector centrality* formalizes the notion that a connection to an important node counts more than a connection to a less important node.

10 $${C}_{E}({x}_{i})=\frac{1}{\lambda }\sum _{j\in N({x}_{i})}^{n}{v}_{j}\,,$$

where *λ* is the largest eigenvalue of the (possibly weighted) adjacency matrix *W*, *v*_*j*_ is the *j*^th^ element of the corresponding eigenvector, and *N*(*x*_*i*_) is the set of nodes that are adjacent to *x*_*i*_.

## Varying the True Edge Weight

**Figure 7:**
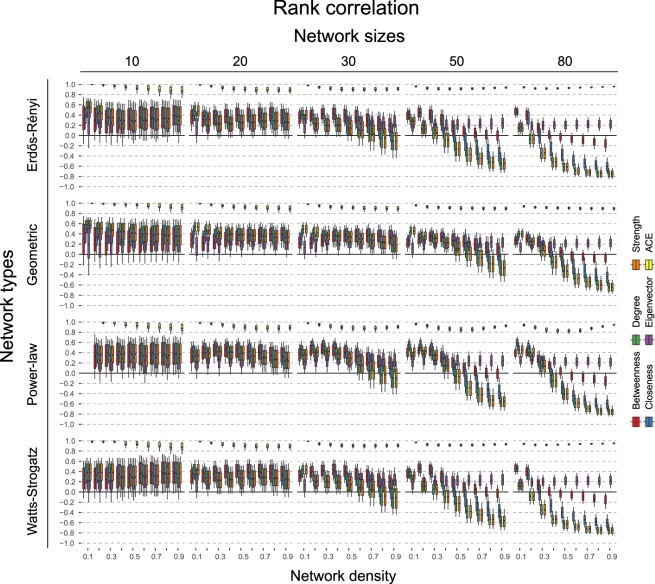
Shows the rank-based correlation of the information theoretic causal effect measure *CE*_*KL*_ with other measures across varying types of networks, number of nodes, and connectivity levels. Error bars denote 10% and 90% quantiles across the 500 simulations. True edge weight is drawn from a Normal (0.0, 0.5) distribution. Black lines indicate the zero correlation level.

**Figure 8:**
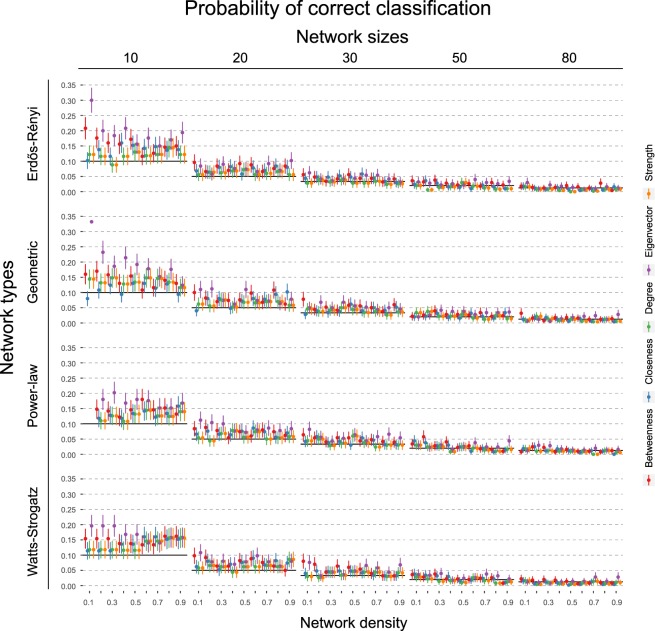
Shows probability of choosing the node with the highest causal effect when using the node with the highest respective centrality measure. Black horizontal lines indicate chance level. Error bars denote 95% confidence intervals across the 500 simulations. True edge weight is drawn from a Normal (0.0, 0.5) distribution.

In the main text, we presented simulations based on DAGs where the true edge weight is drawn from a Normal (0.3, 0.5) distribution. We noticed that the larger the true edge weight, the earlier the phenomena reported take place. For example, if we draw the true edge weights from a Normal (0.0, 0.5) distribution, the negative trend of the correlation as a function of network density only becomes pronounced with a node size of 80; see Figs 7 and 8. Moreover, eigenvector centrality does not show a steep positive correlation. We suspect that this will require even larger graphs. We do not have a strong conviction as to what edge weight value is most likely in the real world–this will depend on the research area–but suggest that Normal (0.3, 0.5) is more likely than Normal (0.0, 0.5). Note that we cannot simulate graphs larger than 50 nodes with an edge weight of 0.3 as the resulting covariance matrix becomes singular.

A notable observation is further that the KL based causal effect measure constitutes a ‘winner-take-all’ mechanism in that only very few nodes, sometimes only a single one, score high on measures of causal effect; the rest flattens out quickly. In contrast, the (total) average causal effect shows a natural linear trend. This has little influence on our results. However, if one were to classify not the top but, say, the first five nodes, the KL based causal effect measure would be an inappropriate target as singles out only the winning node.

## Data Availability

The code for the simulations and the Shiny app is available from https://github.com/fdabl/Centrality-Causality.
